# Clinoptilolite-Based Adsorbents for Paracetamol Removal

**DOI:** 10.3390/molecules30234506

**Published:** 2025-11-22

**Authors:** Szymon Wójcik, Katarzyna Fendrych, Włodzimierz Mozgawa, Magdalena Król

**Affiliations:** Faculty of Materials Science and Ceramic, AGH University of Krakow, 30 Mickiewicza Av., 30-059 Kraków, Poland; szwojcik@agh.edu.pl (S.W.); fendrych@agh.edu.pl (K.F.); mozgawa@agh.edu.pl (W.M.)

**Keywords:** natural zeolite, drug sorption, structure modification, organo-zeolite

## Abstract

This study investigates the adsorption of paracetamol from aqueous solutions using natural clinoptilolite and its modified forms. The raw zeolite (p-CLI) was converted into its protonic (H-CLI) and organo-modified (o-CLI) counterparts through ammonium exchange and calcination, and treatment with hexadecyltrimethylammonium bromide (HDTMA-Br), respectively. The materials were characterized by XRD, FT-IR, and SEM analyses. XRD confirmed that the clinoptilolite crystalline framework was preserved after both modifications, while FT-IR and SEM revealed partial removal of exchangeable cations in H-CLI and the formation of an HDTMA-derived organic layer on the external surface of o-CLI. Adsorption experiments were carried out under batch conditions at initial paracetamol concentrations of 0.5–10 mg/L, and equilibrium paracetamol concentrations were determined using differential pulse voltammetry (DPV). The raw clinoptilolite exhibited negligible adsorption capacity (<0.10 mg/g) due to its hydrophilic surface and microporous framework, which limit interaction with neutral organic molecules. Conversion to the protonic form slightly enhanced the adsorption performance (~0.15 mg/g), while HDTMA modification resulted in a modest additional increase (~0.25 mg/g), attributed to the formation of hydrophobic and organophilic surface sites. Overall, the results indicate that surface functionalization can improve the affinity of clinoptilolite toward weakly polar pharmaceuticals; however, the adsorption capacities remain limited. The novelty of this work lies in combining voltametric quantification with a direct comparison of proton-exchanged and surfactant-modified clinoptilolite to elucidate how specific structural and surface changes influence paracetamol uptake.

## 1. Introduction

Paracetamol (PCT), also known as acetaminophen (N-acetyl-p-aminophenol), is one of the most widely used analgesic and antipyretic drugs worldwide, frequently available over the counter [1,2,3,4,5,6]. Due to its extensive application in recent years, particularly for alleviating symptoms of influenza and the common cold, as well as its increased use during the COVID-19 pandemic, global consumption of PCT has steadily risen [3,6,7]. Paracetamol is now recognized as an emerging organic micropollutant and has been detected in drinking water, surface waters, municipal wastewater, and hospital effluents, where concentrations may exceed 1300 µg/L [2,3,6,7,8,9,10,11]. Although generally considered safe at therapeutic doses, PCT and its degradation products (such as 4-aminophenol) pose risks to aquatic organisms and may contribute to hepatotoxicity and nephrotoxicity [1,7,11,12,13].

Due to its high solubility, stability, and limited removal in conventional wastewater treatment plants, developing efficient and environmentally friendly methods for eliminating paracetamol from aqueous systems has become an important research priority [14,15,16,17,18]. While advanced oxidation processes (AOPs) can effectively degrade PCT, their high operational cost, energy demand, and the possible formation of toxic by-products limit their applicability on a larger scale [2,10,17]. Adsorption represents a more practical and cost-effective method, offering mild operating conditions, no harmful reaction products, and straightforward scalability. Numerous adsorbents—such as activated carbon, biopolymers, zeolites, and zeolite-based composites—have shown high efficiency in removing pharmaceuticals and other micropollutants from water [7,19,20,21,22,23,24,25,26,27,28].

An essential aspect of studying pharmaceutical pollution is the accurate quantification of paracetamol in aqueous systems. Traditional analytical methods, such as high-performance liquid chromatography (HPLC) or gas chromatography (GC), although highly reliable, are time-consuming, require costly instrumentation, and involve multi-step sample preparation [10,29,30]. For this reason, electroanalytical techniques—particularly voltammetric approaches—have gained increasing attention as fast, sensitive, and cost-effective alternatives [10,30,31,32,33,34]. Differential pulse voltammetry (DPV) enables low detection limits and straightforward instrumentation, making it suitable for monitoring paracetamol in adsorption experiments without complex extraction procedures. This analytical advantage provides a practical complement to the adsorption studies performed in the present work.

Clinoptilolite, a naturally occurring zeolite from the heulandite group, has recently attracted significant attention as an efficient and environmentally friendly adsorbent for the removal of organic and inorganic contaminants from water [35,36]. Owing to its high cation-exchange capacity, large surface area, thermal and chemical stability, and well-defined microporous structure, clinoptilolite enables effective adsorption of various pollutants, including heavy metals, ammonium ions, dyes, and pharmaceuticals. Moreover, it is abundant in nature and relatively inexpensive compared to synthetic zeolites or activated carbon, which makes it a promising material for large-scale wastewater treatment applications. Previous studies have demonstrated that the adsorption efficiency of clinoptilolite can be further enhanced through chemical or thermal modification, for instance, by acid activation or ion exchange with transition metals, leading to improved affinity toward organic molecules [37]. However, systematic investigations of paracetamol adsorption on natural or modified clinoptilolite remain limited.

The present study aims to investigate the adsorption behavior of paracetamol from aqueous solutions onto natural clinoptilolite (parent; p-CLI), its hydrogen-exchanged form (H-clinoptilolite; H-CLI), and organo-modified clinoptilolite with hexadecyltrimethylammonium bromide (HDTMA-Br) (o-CLI). The influence of initial paracetamol concentration and zeolite surface modification on adsorption capacity and efficiency was evaluated. The obtained results provide insight into the role of surface chemistry in removing pharmaceutical contaminants from aqueous environments. The protonic form (H-CLI) was selected because replacement of native exchangeable cations with protons can modify the surface acidity and enhance interactions with organic molecules through hydrogen bonding or electrostatic effects [38]. In contrast, HDTMA modification is a well-established approach for producing organo-zeolites with hydrophobic and organophilic external surfaces. Such materials show improved affinity toward weakly polar or neutral pharmaceutical compounds, making them suitable candidates for evaluating the adsorption behavior of paracetamol [39]. In addition to offering a comparative perspective, this study provides a critical assessment of the suitability of clinoptilolite-based materials for removing paracetamol from water. Despite measurable changes in surface chemistry induced by proton exchange and HDTMA treatment, our findings demonstrate that the adsorption capacities remain low. This indicates that the intrinsic microporosity and hydrophilic framework of clinoptilolite impose fundamental limitations on its affinity toward weakly polar drug molecules. The present work, therefore, not only examines modification strategies but also delineates their practical boundaries, highlighting the need for alternative or more advanced functionalization approaches. Moreover, differential pulse voltammetry is employed as a highly sensitive quantification method, providing an analytical approach rarely used in adsorption studies involving natural zeolites.

The aims of this work were as follows:

- To prepare and characterize natural clinoptilolite, its proton-exchanged form, and an HDTMA-modified organo-zeolite;
- To evaluate how these modification strategies influence the structural and surface properties of clinoptilolite;
- To investigate the adsorption of paracetamol from aqueous solutions onto the three zeolite forms;
- To determine the effect of surface chemistry on adsorption performance;
- To critically assess the practical limitations of clinoptilolite-based materials as adsorbents for weakly polar pharmaceuticals.

## 2. Results and Discussion

### 2.1. Materials Characterization

The natural zeolite used in this study was clinoptilolite obtained from the Karpatianian deposit (Slovakia). The chemical composition of the zeolite, determined by X-ray fluorescence (XRF), is presented in Table 1.

**Figure 1:**
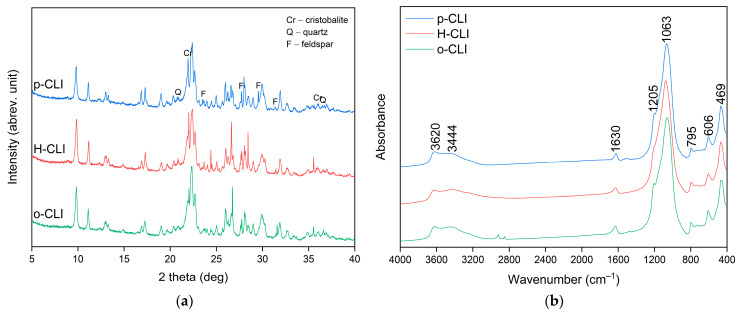
XRD patterns (**a**) and FT-IR spectra (**b**) of the raw (p-CLI), protonic (H-CLI), and HDTMA-modified (o-CLI) clinoptilolite samples.

The XRD patterns of the raw (p-CLI), protonic (H-CLI), and HDTMA-modified (o-CLI) clinoptilolite samples are shown in Figure 1a. All diffractograms exhibit the characteristic reflections of clinoptilolite at approximately 2θ = 9.8°, 11.2°, 22.4°, 26.1°, and 30.0°, confirming the preservation of the heulandite-type crystal structure after the modification processes [36,40]. Minor reflections corresponding to quartz (Q), feldspar (F), and cristobalite (Cr) were also identified, which is consistent with the natural mineral impurities typically associated with clinoptilolite deposits. No additional diffraction peaks were detected after ion exchange or surfactant treatment, indicating that neither the ion exchange and calcination used to obtain the H-form nor the subsequent HDTMA^+^ modification caused any structural degradation or formation of new crystalline phases.

The FT-IR spectra of the raw (p-CLI), protonic (H-CLI), and organo-modified (o-CLI) clinoptilolite samples are presented in Figure 1b. All spectra exhibit the characteristic absorption bands of the clinoptilolite framework. The intense band at about 1063 cm^−1^ corresponds to the asymmetric stretching vibrations of Si–O–Si and Si–O–Al bonds, while those at approximately 795 and 606 cm^−1^ are assigned to symmetric stretching and bending modes, respectively. The band near 469 cm^−1^ originates from the bending vibrations of Si–O–Si(Al) linkages [41]. The broad absorption centered at about 3440 cm^−1^, together with the band at 1630 cm^−1^, is attributed to the stretching and bending vibrations of adsorbed water molecules. The weak peak at 3620 cm^−1^ observed in the raw sample corresponds to structural -OH groups associated with octahedral cations (e.g., Al–OH or Fe–OH). After ion-exchange and calcination (H-CLI), a decrease in the intensity of the hydroxyl and water-related bands is evident, confirming partial dehydration and the removal of exchangeable cations. In the spectrum of the organo-modified sample (o-CLI), the overall framework bands remain unchanged, indicating the preservation of the zeolite crystalline structure. However, subtle changes in band intensity and broadening are observed, along with the appearance of new absorption bands in the 2850–2950 cm^−1^ region, corresponding to the aliphatic C–H stretching vibrations of -CH_2_ and -CH_3_ groups from the surfactant. These features confirm the successful attachment of organic species onto the zeolite surface.

To support these subtle spectral differences, elemental SCHN analysis was performed. The parent clinoptilolite contained only 0.35 wt% carbon, while the proton-exchanged sample showed a comparable value (0.38 wt%), indicating that no organic species were introduced during protonation. In contrast, the HDTMA-modified sample exhibited a marked increase to 3.38 wt% carbon, accompanied by higher hydrogen and nitrogen contents, which directly reflects the presence of the quaternary ammonium surfactant on the external surface.

**Figure 2:**
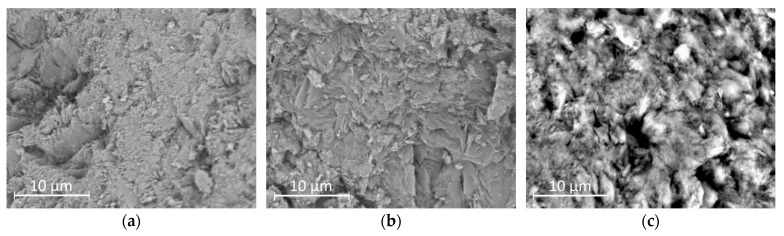
SEM micrographs of clinoptilolite samples: raw clinoptilolite (p-CLI) (**a**); H-CLI (**b**); and HDTMA-modified clinoptilolite (o-CLI) (**c**).

Scanning electron microscopy (SEM) was used to examine the surface morphology of the natural clinoptilolite and its modified forms (H-CLI and o-CLI). The micrographs revealed distinct morphological differences among the samples, reflecting the effects of chemical treatment and surface modification (Figure 2).

Raw clinoptilolite (Ca/K form; Figure 2a) exhibited a well-developed crystalline structure typical of heulandite-type zeolites. The particles were irregularly shaped and composed of aggregated plate-like and prismatic crystals with sharp edges and clearly visible cleavage planes. The surfaces appeared relatively smooth and compact, indicating minimal amorphous or fine material coverage. Some minor debris and fine particles were observed in the interparticle spaces, likely corresponding to clay impurities or secondary mineral phases such as quartz or feldspar. These features are consistent with the morphology of natural clinoptilolite reported in previous studies [36,42].

**Figure 3:**
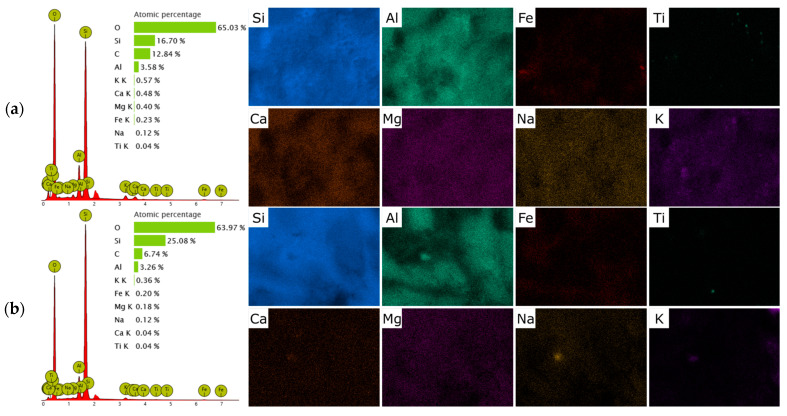

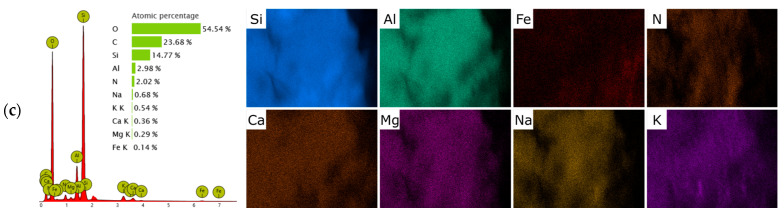
SEM–EDS elemental mapping of raw clinoptilolite (p-CLI) (**a**); H-CLI (**b**); and HDTMA-modified clinoptilolite (o-CLI) (**c**).

After preparing H-form (Figure 2b), the overall morphology of the particles remained unchanged, confirming that the treatment did not significantly alter the crystalline framework. However, the surface became slightly rougher and more porous, with evidence of partial leaching of exchangeable cations (Ca^2+^, K^+^, Na^+^) and partial dissolution of amorphous material (Figure 3). This increased roughness and the possible generation of mesopores could contribute to the improved sorption performance observed for the H-form compared to the raw zeolite. In contrast, the HDTMA-modified clinoptilolite (Figure 2c) displayed a distinctly different surface texture. The SEM images showed that the originally sharp crystal edges were partially covered by a thin, continuous layer attributed to the deposited surfactant. The surface appeared smoother and less angular, with some areas showing agglomerated organic films or micellar aggregates. No significant structural collapse or fragmentation was observed, indicating that the modification preserved the overall integrity of the zeolite framework. The continuous surfactant layer introduces hydrophobic and organophilic sites on the zeolite surface, which enhance interactions with weakly polar molecules such as paracetamol. Although the surface appears smoother compared to the H-form, the preservation of overall porosity and the presence of micellar aggregates should provide accessible adsorption sites.

### 2.2. Adsorption Studies Determined by Voltammetric Analysis

This section presents the adsorption performance of the raw (p-CLI), protonic (H-CLI), and organo-modified (o-CLI) clinoptilolite samples toward paracetamol, determined using voltammetric techniques. The adsorption experiments were conducted under identical conditions to evaluate the influence of surface modification on the affinity of clinoptilolite for the target compound. Differential pulse voltammetry (DPV) was employed for the quantitative determination of paracetamol concentration in aqueous solutions before and after the adsorption process, owing to its high sensitivity and selectivity toward phenolic compounds [43]. The obtained results allow for a comparative assessment of sorption efficiency and provide insight into the effect of surface chemistry on the interaction between paracetamol molecules and the zeolite surface.

The results of the initial (*C*_0_) and equilibrium (*C_e_*) concentrations of paracetamol, along with the calculated equilibrium adsorption capacities (*q_e_*), are summarized in Table 2. All recorded concentrations fall within the linear range of the analytical method. The high correlation coefficients (R > 0.9960) observed for all data sets confirm the repeatability and reliability of the measurements.

**Figure 4:**
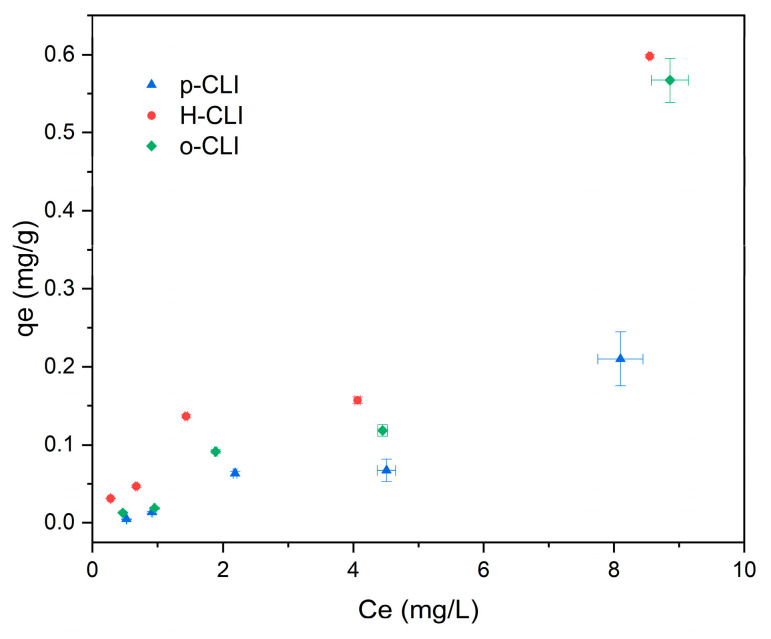
Relationship between equilibrium concentration (*C_e_*) and equilibrium adsorption capacity (*q_e_*) of paracetamol.

The adsorption isotherms presented in Figure 4 show a clear dependence of the equilibrium adsorption capacity (*q_e_*) on the equilibrium concentration of paracetamol (*C_e_*). Among the tested materials, the protonic form of clinoptilolite (H-CLI) exhibited the highest adsorption capacity, followed by the organo-modified sample (o-CLI), while the parent material (p-CLI) showed the lowest uptake. This indicates that the surface modification through ammonium exchange and subsequent calcination substantially enhances the affinity of clinoptilolite toward paracetamol, likely due to an increase in the number of accessible acidic sites and improved surface polarity. The slight improvement observed for o-CLI suggests that the presence of surfactant molecules may contribute to adsorption through hydrophobic interactions, but the effect is less pronounced than that of protonation. A slight plateau observed in the mid-concentration range, followed by a secondary increase, suggests the coexistence of multiple adsorption mechanisms or gradual activation of additional sites at higher concentrations. Overall, the shape of the curves confirms a low but measurable affinity of clinoptilolite-based materials toward paracetamol, with only moderate enhancement achieved through surface treatment.

The maximum adsorption capacities obtained for p-CLI, H-CLI, and o-CLI fall within the range typically reported for clinoptilolite-based materials. Previous studies have shown that natural clinoptilolite exhibits minimal affinity toward paracetamol, with reported capacities below 0.3 mg/g, depending on experimental conditions [40,42]. The maximum sorption capacity observed in this study was in the range of 0.013–0.150 mg/g, confirming that the natural zeolite in its parent form is practically ineffective for the removal of paracetamol from aqueous solutions. HDTMA-modified zeolites generally show improved adsorption, although values remain below 1 mg/g due to the persistence of steric and structural constraints [42,44]. These literature data are in agreement with our results, indicating that the intrinsic microporous, strongly hydrophilic framework of clinoptilolite fundamentally restricts the uptake of weakly polar molecules such as paracetamol. By comparison, activated carbon, biochar, graphene-derived materials, and MOFs typically achieve much higher capacities, ranging from 20 to 300 mg/g [45], highlighting the practical limitations of clinoptilolite as a sorbent for paracetamol.

The limited adsorption performance can be attributed to both the physicochemical properties of the clinoptilolite surface and the molecular characteristics of paracetamol. Natural clinoptilolite is an aluminosilicate framework with a negatively charged lattice compensated by exchangeable cations such as Ca^2+^, K^+^, and Na^+^. Consequently, its surface is strongly hydrophilic and polar, favoring the adsorption of hydrated inorganic cations rather than neutral organic molecules. Paracetamol (acetaminophen), on the other hand, is a neutral molecule at typical environmental pH (pK_a_ ≈ 9.4), with only moderate polarity (log K_o_w ≈ 0.46) [44]. Therefore, electrostatic interactions and ion-exchange processes, which dominate the sorption of ionic species on zeolites, do not contribute to its retention on the raw mineral surface [40]. This interpretation is supported indirectly by FT-IR spectra (Figure 1b) and EDS analysis (Figure 3), which show the dominant presence of hydrated extra-framework cations in the parent material and no evidence of functional groups capable of interacting specifically with aromatic or amide moieties.

Moreover, the microporous structure of clinoptilolite imposes an additional steric limitation [46]. The pore apertures of clinoptilolite (~0.4 nm) are smaller than the molecular dimensions of paracetamol (~0.6 nm), preventing the molecule from entering the internal channels of the zeolite framework. As a result, adsorption can occur only on the external surface, which has a relatively small specific area and few active sites capable of interacting with aromatic or hydrophobic moieties. This explains the negligible adsorption observed even after prolonged contact times.

Negatively charged, hydrophilic surface and narrow pore system of clinoptilolite restrict access and interaction with the paracetamol molecule. Effective removal of this compound thus requires surface modification to create new adsorption sites and alter the surface polarity. This conclusion aligns with contemporary research emphasizing the need for zeolite functionalization when targeting the sorption of neutral, weakly polar pharmaceuticals from aqueous environments [40,42].

Following the ion-exchange treatment with ammonium ions and subsequent calcination, a noticeable increase in the sorption capacity toward paracetamol was observed. This improvement can be explained by several structural and surface modifications that occur during the conversion of the initial clinoptilolite into its protonic (H-) form. Hydrogen-form clinoptilolite is characterized by the presence of Brønsted acid sites and a partially dehydroxylated surface. These acidic hydroxyl groups (Si–OH–Al) can form sp hydrogen bonding with the hydroxyl and amide groups of the paracetamol molecule, thereby increasing the overall affinity between the adsorbate and the adsorbent surface [42,47]. In addition, the removal of extra-framework cations and water molecules during calcination enhances the specific surface area and pore accessibility of the zeolite. The elimination of blocking cations opens up previously inaccessible micropores and increases the fraction of mesoporous regions, facilitating the diffusion of paracetamol molecules toward the internal adsorption sites. Although the intrinsic pore size of clinoptilolite (~0.4 nm) still limits complete penetration of the molecule (~0.6 nm), the increased availability of external and near-surface sites after calcination contributes to a measurable rise in sorption capacity.

Another important factor is the change in surface polarity and acid-base character. While the parent zeolite exhibits a predominantly hydrophilic and electrostatically negative surface, the H-form is less hydrated and more amphoteric, capable of forming weak hydrogen-bonding or donor-acceptor interactions with the aromatic and amide groups of paracetamol. This enhanced interaction is consistent with the moderate polarity of the molecule and its ability to act both as a hydrogen-bond donor and acceptor. Consequently, adsorption in the H-form is governed not by ion-exchange mechanisms but by specific surface interactions between the aromatic ring of paracetamol and the siliceous surface. Although no direct acidity measurements (such as NH_3_-TPD or potentiometric titration) were performed, the observed removal of exchangeable cations (Figure 3) and the appearance of characteristic Si–OH–Al features in FT-IR spectra (Figure 1b) provide accepted indirect evidence of increased Brønsted acidity and reduced surface hydration in H-CLI.

Following the surface modification with hexadecyltrimethylammonium bromide (HDTMA-Br), the sorption capacity of clinoptilolite toward paracetamol showed only a slight improvement compared with the initial material. Such a modest increase suggests that the modification did not substantially alter the adsorption mechanism or the surface affinity toward the paracetamol molecules. Similar trends have been reported for other weakly polar organic compounds, where HDTMA-modified zeolites enhanced sorption only marginally, particularly when the molecular size of the adsorbate exceeded the zeolite pore dimensions [40,47]. In theory, surfactant modification with HDTMA^+^ introduces a hydrophobic organic phase on the external surface of the zeolite, converting it from hydrophilic to organophilic. The positively charged quaternary ammonium head groups anchor to the negatively charged aluminosilicate framework, while the long alkyl chains extend outward, forming a bilayer or hemimicellar arrangement [48]. This configuration is expected to facilitate the adsorption of neutral and weakly polar organic molecules through van der Waals and π–π interactions. However, in the present case, several factors likely limited the effectiveness of this modification.

To quantitatively describe the adsorption behavior of paracetamol on the tested clinoptilolite materials, the experimental data were fitted to the Langmuir and the Freundlich isotherm models. The Langmuir model assumes monolayer adsorption on a homogeneous surface with a finite number of identical sites, while the Freundlich model accounts for adsorption on heterogeneous surfaces and describes multilayer formation. Both models were applied to provide insights into the adsorption capacity and intensity.

The fitting results are summarized in Table 3. The Langmuir model showed very poor correlation with the experimental data for all three materials (R^2^ ranging from 0.0715 to 0.4147), and in the case of the organo-modified clinoptilolite (o-CLI), a negative *q_max_*, indicating that monolayer adsorption on a homogeneous surface does not describe the system. Poor Langmuir fitting has also been observed in previous studies of paracetamol adsorption on clay minerals and natural zeolites, where the heterogeneous structure of aluminosilicate surfaces prevents the formation of an ideal monolayer [19]. In contrast, the Freundlich model provided a much better fit (R^2^ > 0.92), confirming the heterogeneous nature of the adsorbent surfaces and the likely occurrence of multilayer adsorption. This trend is consistent with the literature reports indicating that Freundlich-type behavior is typical for the adsorption of pharmaceuticals [49].

The H-CLI sample exhibited the highest *K_F_* (0.0786 mg/g·(L/mg)^1/*n*^) and favorable adsorption (1/*n* = 0.8153), while the organo-modified o-CLI showed lower capacity (*K_F_* = 0.0284) and less favorable adsorption (1/*n* = 1.2584). The adsorption intensity parameter (1/*n*) values were below 1 for p-CLI and H-CLI, reflecting favorable adsorption, while the value exceeding 1 for o-CLI suggests less favorable adsorption conditions under the studied concentration range. This indicates that protonation significantly enhances paracetamol adsorption through increased surface acidity, whereas surfactant modification has a moderate effect, likely via hydrophobic interactions.

Results demonstrate that surface modification by protonation significantly enhances the adsorption of paracetamol, likely due to the increased number of acidic sites, whereas organo-modification produces only moderate improvement, probably through hydrophobic interactions. The superior fit of the Freundlich model highlights the importance of surface heterogeneity and the coexistence of multiple adsorption mechanisms in these materials. These findings are consistent with previous reports in the literature.

For instance, environmental–pharmaceutical studies on natural zeolites, including clinoptilolite, have shown that Freundlich isotherms often better describe the adsorption of drug molecules such as paracetamol, reflecting a heterogeneous surface and multilayer adsorption [45]. Moreover, adsorption studies of paracetamol on other adsorbents, such as activated carbons, also report that more complex or empirical models (e.g., Sips) fit better than Langmuir, indicating non-ideal monolayer behavior [49,50].

The results obtained in this study demonstrate that the adsorption of paracetamol on both parent and surface-modified clinoptilolite is inherently limited by the physicochemical characteristics of the material and the molecular structure of the adsorbate. Although ion exchange and calcination to obtain the protonic form led to a moderate improvement in adsorption performance, and HDTMA^+^ modification slightly increased affinity toward paracetamol, the overall capacities remained low compared with other adsorbents reported in the literature. Therefore, further optimization of these systems is unlikely to yield substantial gains without more fundamental structural or chemical modification of the zeolite. Future work should consider deeper structural functionalization or composite formation to achieve practical adsorption performance.

## 3. Materials and Methods

### 3.1. Starting Materials

Natural zeolite of Carpathian origin (Slovakia) was used as the base material. The raw clinoptilolite was dried at 105 °C for 24 h, ground, and then sieved to obtain a particle size fraction with a diameter below 0.1 mm. Three forms of zeolite were prepared: the parent (untreated) form, the hydrogen-exchanged form (H-clinoptilolite), and the HDTMA-modified form (organo-zeolite).

The hydrogen form was obtained via double ion exchange using a 0.1 M NH_4_NO_3_ solution at 60 °C, with a solid-to-liquid ratio of 1 g:20 mL. Each exchange step lasted 2 h. The material was subsequently washed three times with distilled water, dried at 105 °C, and calcined at 450 °C for 5 h to convert the ammonium form to the hydrogen form.

To obtain organo-modified clinoptilolite, zeolite was first converted into its sodium form and then treated with a 30 mM aqueous solution of hexadecyltrimethylammonium bromide (HDTMA-Br) at 30 °C for 24 h under continuous stirring. The resulting suspension was centrifuged, repeatedly washed with distilled water to remove excess surfactant, and dried at 105 °C.

All three zeolite samples (parent, H-, and organo-forms) were subjected to structural and physicochemical characterization, including X-ray diffraction (XRD), Fourier-transform infrared spectroscopy (FT-IR), scanning electron microscopy (SEM), and elemental composition analysis (XRF).

### 3.2. Characterization Methods

The elemental composition of the clinoptilolite samples before and after modifications was analyzed by wavelength-dispersive X-ray fluorescence spectroscopy (WD-XRF). Measurements were carried out using an Axios Max spectrometer (PANalytical, Malvern, UK) equipped with a 4 kW Rh anode X-ray tube. Data acquisition was performed in standard analytical mode with the Omnian software package (PANalytical, Malvern, UK).

The crystalline phase composition was determined by X-ray diffraction (XRD) using a Philips X’Pert diffractometer (PANalytical, Malvern, UK) operating with CuKα radiation (λ = 1.5418 Å). Diffraction patterns were collected in the 2θ range of 5–90°, with a step interval of 0.007° and a total scan time of approximately two hours. Phase identification was conducted using the Match! 4 software in conjunction with reference data from the Crystallography Open Database (COD).

Fourier transform infrared (FT-IR) spectra were recorded using a Bruker VERTEX 70v Fourier-transform infrared spectrometer (Bruker, Billerica, MA, USA) under vacuum conditions. Spectra were obtained in the range of 4000–400 cm^−1^ in absorbance mode using the conventional KBr pellet method. Each spectrum represented the average of 128 scans at a spectral resolution of 4 cm^−1^. Before analysis, all spectra underwent linear baseline correction.

The morphology of the samples was examined by scanning electron microscopy (SEM). Before observation, the powders were sputter-coated with a thin layer of gold. Imaging in backscattered electron (BSE) mode was performed on a Phenom XL desktop SEM (ThermoFisher Scientific, Waltham, MA, USA) operating at 10 kV. The microscope was equipped with an energy-dispersive X-ray spectroscopy (EDS) detector, which was employed to obtain local chemical composition data.

### 3.3. Adsorption Experiments

Batch adsorption experiments were conducted to evaluate the ability of natural, hydrogen-exchanged, and HDTMA-modified clinoptilolite to remove paracetamol from aqueous solutions. Adsorption tests were performed using initial paracetamol concentrations of 0.5, 1.0, 2.0, 5.0, and 10.0 mg/L. For each experiment, 0.10 g of zeolite was added to 10 mL of paracetamol solution, corresponding to a solid-to-liquid ratio of 10 g/L. The suspensions were agitated on a laboratory shaker at ambient temperature for 24 h to ensure equilibrium conditions. The natural pH of the solutions was maintained throughout the process, without external adjustment. After equilibration, the samples were centrifuged to separate the solid and liquid phases. The supernatant was collected for voltammetric determination of the residual paracetamol concentration (*C_e_*), while the solid residue was washed three times with distilled water and retained for further structural analysis. All adsorption tests were carried out in triplicate under identical conditions to ensure reproducibility. The amount of adsorbed paracetamol (*q_e_*, mg/g) was calculated based on the concentration difference between the initial (*C*_0_) and equilibrium (*C_e_*) solutions according to the mass balance equation:

(1) $$q_{e}=\frac{(C_{0}-C_{e})V}{m},$$

where *V* (L) is the solution volume and *m* (g) is the mass of adsorbent.

To describe the adsorption behavior, the equilibrium data were further analyzed using Langmuir (2) and Freundlich (3) isotherm models:

(2) $$q_{e}=\frac{q_{max}K_{L}C_{e}}{1+K_{L}C_{e}},$$

(3) $$q_{e}=K_{F}C_{e}^{1/n}.$$

Langmuir parameters (maximum adsorption capacity, *q_max_*, and Langmuir constant, *K_L_*) and Freundlich parameters (adsorption capacity constant, *K_F_*, and intensity parameter, 1/*n*) were determined by linear regression of the transformed equations. The goodness of fit for each model was evaluated using the correlation coefficient (R^2^).

### 3.4. Differential Pulse Voltammetry

Electrochemical experiments were conducted using an M161 electrochemical analyzer in combination with an M164 three-electrode stand (MTM-ANKO, Kraków, Poland), enabling current acquisition at a frequency of 1 kHz. The experimental setup consisted of a conventional three-electrode configuration, comprising a glassy carbon (GC) working electrode, a double-junction Ag|AgCl|KCl (3 M) reference electrode with a silver wire, and a platinum wire auxiliary electrode. Before each measurement, the surface of the GC electrode was mechanically prepared by polishing with alumina slurries of decreasing particle sizes to ensure a clean and reproducible surface.

Data acquisition and preliminary signal processing were carried out using the EAQt software package (Krakow, Poland), specifically designed for integration with the applied instrumentation. Furthermore, to ensure reproducibility and accuracy of the measurements, the EACfg software was employed. This program, fully compatible with the measurement system, enabled automated data collection within precisely defined temporal parameters.

The voltammetric determination of paracetamol was performed under optimized experimental conditions [43]. To ensure high analytical precision and reproducibility, all voltammograms were recorded automatically. An appropriate supporting electrolyte was used to enhance the solution conductivity. The most satisfactory responses were obtained using a mixture of 2.5 mL of 0.1 M phosphate buffer (pH 6.0) and 2.5 mL of the sample solution.

**Figure 5:**
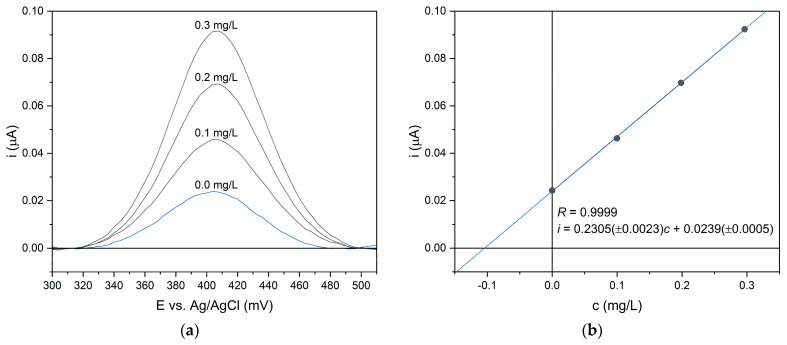
Differential pulse voltammograms (**a**) and corresponding calibration curve (**b**) obtained for paracetamol in aqueous solution under optimized measurement conditions.

The voltammograms were recorded within the potential window of 100–600 mV, using a potential step of 4 mV. Both the waiting and sampling times were set to 20 ms. To improve electrode performance and surface stability, measurements were carried out in a cyclic mode. Each recorded peak exhibited a symmetrical shape with a baseline parallel to the potential axis, indicating stable electrode behavior and high signal quality. Therefore, baseline correction using polynomial approximation did not significantly affect the analytical parameters. For all measurements, the peak potential was observed at 412 mV (Figure 5a). Quantification of paracetamol was carried out using the standard addition method (Figure 5b).

## 4. Conclusions

This study evaluated the adsorption of paracetamol from aqueous solutions onto natural clinoptilolite (p-CLI), its protonic form (H-CLI), and organo-modified form (o-CLI) using differential pulse voltammetry (DPV). The experimental results demonstrated that the adsorption efficiency strongly depends on the surface chemistry of the zeolite.

The parent clinoptilolite exhibited a very low adsorption capacity, with the maximum equilibrium value of approximately 0.15 mg/g, confirming its limited affinity for weakly polar organic molecules. Conversion to the hydrogen form (H-CLI) significantly enhanced the adsorption performance, reaching a maximum of 0.60 mg/g, which can be attributed to the generation of Brønsted acid sites and improved accessibility of surface pores. Modification with hexadecyltrimethylammonium bromide (HDTMA-Br) yielded only a modest increase, with a maximum capacity of 0.57 mg/g, suggesting that surfactant-derived hydrophobic layers provide limited benefit for paracetamol uptake. These findings confirm that the adsorption of paracetamol on clinoptilolite-based materials is mainly governed by surface polarity and pore accessibility rather than by hydrophobic interactions. While surface protonation effectively enhances sorption, organophilization by cationic surfactants does not substantially improve adsorption efficiency for moderately polar pharmaceuticals such as paracetamol.

Clinoptilolite remains a low-cost, stable, and environmentally friendly adsorbent with limited performance toward paracetamol removal. Further enhancement could be achieved through composite formation with carbonaceous phases or metal-ion exchange to increase surface reactivity. Such approaches may offer a promising route toward developing zeolite-based materials with higher affinity for pharmaceutical pollutants in water treatment applications.

## Figures and Tables

**Table 1:** Chemical composition of initial clinoptilolite.

Oxides	SiO_2_	TiO_2_	Al_2_O_3_	Fe_2_O_3_	CaO	MgO	K_2_O	Na_2_O	Others
Ratio by weight, (%)	75.46	0.33	11.89	2.34	4.45	0.54	3.87	0.83	0.29

**Table 2:** Initial (*C*_0_) and equilibrium (*C_e_*) concentrations of paracetamol and calculated equilibrium adsorption capacities (*q_e_*) for the raw (p-CLI), protonic (H-CLI), and organo-modified (o-CLI) clinoptilolite samples.

Name	*C*_0_, mg/L	*R* *	*C_e_*, mg/L	*R* *	*q_e_*, mg/g
p-CLI	0.57 ± 0.07	0.9990	0.52 ± 0.01	0.9999	0.013 ± 0.001
	1.05 ± 0.13	0.9987	0.92 ± 0.01	0.9997	0.013 ± 0.001
	2.81 ± 0.09	0.9999	2.19 ± 0.03	0.9997	0.063 ± 0.003
	5.18 ± 0.13	1.0000	4.51 ± 0.14	0.9962	0.067 ± 0.014
	10.20 ± 0.11	0.9991	8.70 ± 0.28	0.9999	0.150 ± 0.035
H-CLI	0.59 ± 0.02	0.9997	0.28 ± 0.01	0.9999	0.031 ± 0.001
	1.14 ± 0.09	0.9989	0.68 ± 0.04	1.0000	0.047 ± 0.001
	2.80 ± 0.22	0.9985	1.43 ± 0.14	1.0000	0.137 ± 0.002
	5.64 ± 0.16	0.9999	4.07 ± 0.10	0.9999	0.157 ± 0.004
	14.53 ± 0.30	0.9999	8.55 ± 0.23	0.9999	0.598 ± 0.002
o-CLI	0.59 ± 0.02	0.9997	0.47 ± 0.02	0.9997	0.013 ± 0.001
	1.14 ± 0.09	0.9989	0.95 ± 0.06	0.9995	0.019 ± 0.001
	2.80 ± 0.22	0.9985	1.89 ± 0.13	0.9988	0.091 ± 0.002
	5.64 ± 0.16	0.9999	4.45 ± 0.16	0.9999	0.119 ± 0.008
	14.53 ± 0.30	0.9999	8.86 ± 0.53	0.9996	0.567 ± 0.028

* *R*—correlation coefficient of the voltammetric calibration measurements, indicating linearity.

**Table 3:** Langmuir and Freundlich isotherm parameters for the adsorption of paracetamol on different clinoptilolite materials. Langmuir model parameters: maximum adsorption capacity (*q_max_*) and Langmuir constant (*K_L_*); Freundlich model parameters: adsorption capacity constant (*K_F_*) and intensity parameter (1/*n*). R^2^ values indicate the goodness of fit for each model.

Name	Langmuir			Freundlich		
	*q_max_*, mg/g	*K_L_*, L/mg	R^2^	*K_F_*, (mg/g)(L/mg)^1⁄*n*^	1/*n*	R^2^
p-CLI	−0.18	−0.0634	0.3122	0.0136	1.2830	0.9471
H-CLI	1.45	0.0541	0.1274	0.0787	0.8140	0.9250
o-CLI	−0.38	−0.0639	0.4147	0.0282	1.2604	0.9416

## Data Availability

The original contributions presented in this study are included in the article. Further inquiries can be directed to the corresponding author.

## Abbreviations

The following abbreviations are used in this manuscript:

AGCE	activated glassy carbon electrodes
AOP	advanced oxidation process
CPE	carbon paste electrodes
DPV	differential pulse voltammetry
FT-IR	Fourier-transform infrared spectroscopy
GC	gas chromatography
GCE	glassy carbon electrodes
HDTMA	hexadecyltrimethylammonium
HPLC	high-performance liquid chromatography
PCT	Paracetamol
rGO	reduced graphene oxide
SEM	scanning electron microscope
SWV	square wave voltammetry
XRD	X-ray diffraction
XRF	X-ray fluorescence

## References

[1] Bosch M.E., Sánchez A.J.R., Rojas F.S., Ojeda C.B. (2006). Determination of paracetamol: Historical evolution. J. Pharm. Biomed. Anal..

[2] Agarwal N. (2022). Paracetamol—A contaminant of high concern: Existence in environment and adverse effect. PDRAJ.

[3] Vieira Y., Spode J.E., Dotto G.L., Georgin J., Franco D.S.P., dos Reis G.S., Lima E.C. (2024). Paracetamol environmental remediation and ecotoxicology: A review. Environ. Chem. Lett..

[4] Jóźwiak-Bebenista M., Nowak J.Z. (2014). Paracetamol: Mechanism of action, applications and safety concern. Acta Pol. Pharm..

[5] Bertolini A., Ferrari A., Ottani A., Guerzoni S., Tacchi R., Leone S. (2006). Paracetamol: New vistas of an old drug. CNS Drug Rev..

[6] Shih Y.-J., Wu Z.-L., Lin S.-K. (2024). Electrochemical detection of paracetamol using zeolitic imidazolate framework and graphene oxide derived zinc/nitrogen-doped carbon. Sens. Actuators. B Chem..

[7] Skwarczynska-Wojsa A., Puszkarewicz A. (2024). Removal of acetaminophen from aqueous solutions in an adsorption process. Materials.

[8] Bauer A.Z., Swan S.H., Kriebel D., Liew Z., Taylor H.S., Bornehag C.-G., Andrade A.M., Olsen J., Jensen R.H., Mitchell R.T. (2021). Paracetamol use during pregnancy—A call for precautionary action. Nat. Rev. Endocrinol..

[9] Khmiri Y., Attia A., Jallouli N., Chabanon E., Charcosset C., Mahouche-Chergui S., Dammak L., Algieri C., Chakraborty S., Amar R.B. (2025). Synthesis of a cost-effective ZnO/zeolite photocatalyst for paracetamol removal. Emergent Mater..

[10] Montaseri H., Forbes P.B.C. (2018). Analytical techniques for the determination of acetaminophen: A review. TrAC Trends Anal. Chem..

[11] Al-howri B.M., Azha S.F., Shamsudin M.S., Hamid N.A., Alsobaai A.M., Ismail S. (2024). Paracetamol in diverse water sources: Health hazards and treatment efficacy emphasizing adsorption techniques—A review. Int. J. Environ. Sci. Technol..

[12] Yenealem D., Admassie S., Pilli S.R., Motana S., Tibebe D. (2025). Detection and separation of paracetamol and p-aminophenol using activated glassy carbon electrodes. Electrocatalysis.

[13] Weheabby S., Wu Z., Al-Hamry A., Pašti I.A., Anurag A., Dentel D., Tegenkamp C., Kanoun O. (2023). Paracetamol detection in environmental and pharmaceutical samples using multi-walled carbon nanotubes decorated with silver nanoparticles. Microchem. J..

[14] Nguyen T.-K.-T., Nguyen T.-B., Chen C.-W., Chen W.-H., Chen L., Hsieh S., Dong C.-D. (2024). Kumquat peel-derived biochar to support zeolitic imidazole framework-67 (ZIF-67) for enhancing peracetic acid activation to remove acetaminophen from aqueous solution. Environ. Pollut..

[15] Kryuchkova M., Batasheva S., Akhatova F., Babaev V., Buzyurova D., Vikulina A., Volodkin D., Fakhrullin R., Rozhina E. (2021). Pharmaceuticals removal by adsorption with montmorillonite nanoclay. Int. J. Mol. Sci..

[16] Parus A., Gaj M., Karbowska B., Zembrzuska J. (2020). Investigation of acetaminophen adsorption with a biosorbent as a purification method of aqueous solution. Chem. Ecol..

[17] Grela A., Kuc J., Bajda T. (2021). A review on the application of zeolites and mesoporous silica materials in the removal of non-steroidal anti-inflammatory drugs and antibiotics from water. Materials.

[18] Al-rimawi F., Daana M., Khamis M., Karaman R., Khoury H., Qurie M. (2019). Removal of selected pharmaceuticals from aqueous solutions using natural Jordanian zeolite. Arab. J. Sci. Eng..

[19] Serna-Galvis E.A., Arboleda-Echavarría J., Echavarría-Isaza A., Torres-Palma R.A. (2024). Removal and elimination of pharmaceuticals in water using zeolites in diverse adsorption processes and catalytic advanced oxidation technologies—A critical review. Environ. Sci. Pollut. Res..

[20] Pirvu F., Covaliu-Mierlă C.I., Catrina G.A. (2023). Removal of acetaminophen drug from wastewater by Fe_3_O_4_ and ZSM-5 Materials. Nanomaterials.

[21] Fu M., He M., Heijman B., van der Hoek J.P. (2021). Ozone-based regeneration of granular zeolites loaded with acetaminophen. Sep. Purif. Technol..

[22] Araújo A., Soares O.S.G.P., Orge C.A., Gonçalves A.G., Rombi E., Cutrufello M.G., Fonseca A.M., Pereira M.F.R., Neves I.C. (2021). Metal-zeolite catalysts for the removal of pharmaceutical pollutants in water by catalytic ozonation. J. Environ. Chem. Eng..

[23] Yang Y., Liu D., Chen Y., He J., Li Q. (2024). Mechanistic study of highly effective phosphate removal from aqueous solutions over a new lanthanum carbonate fabricated carbon nanotube film. J. Environ. Manag..

[24] Aziz K.H.H., Mustafa F.S., Karim M.A.H., Hama S. (2025). Pharmaceutical pollution in the aquatic environment: Advanced oxidation processes as efficient treatment approaches: A review. Mater. Adv..

[25] Onyekachukwu E., Nesbitt H., Tretsiakova-McNally S., Coleman H. (2025). Low-cost adsorbents for the removal of pharmaceuticals from surface waters. Water.

[26] Hastuti B., Lutfiah S., Hadi S., Utomo S.B., Kamari A. (2025). Development of chitosan/alginate/montmorillonite hydrogel microcomposite as adsorbent for paracetamol removal from waters. Pure Appl. Chem..

[27] Serna-Galvis E.A., Botero-Coy A.M., Arboleda-Echavarría J., Echavarría-Isaza A., Hernández F., Torres-Palma R.A. (2025). Simultaneous elimination of multiple pharmaceuticals in real wastewater effluent using a commercial zeolite and peroxymonosulfate. J. Clean. Prod..

[28] Bajda T., Grela A., Pamuła J., Kuc J., Klimek A., Matusik J., Franus W., Alagarsamy S.K.K., Danek T., Gara P. (2024). Using zeolite materials to remove pharmaceuticals from water. Materials.

[29] Mallah M.A., Sherazi S.T.H., Bhanger M.I., Mahesar S.A., Bajeer M.A. (2015). A Rapid Fourier-transform infrared (FTIR) spectroscopic method for direct quantification of paracetamol content in solid pharmaceutical formulations. Spectrochim. Acta A.

[30] Ahmed M.J., Perveen S., Hussain S.G., Khan A.A., Ejaz S.M.W., Rizvi S.M.A. (2023). Design of a facile, green and efficient graphene oxide-based electrochemical sensor for analysis of acetaminophen drug. Chem. Pap..

[31] Eskezia Ayalew M., Yenealem Ayitegeb D. (2021). Differential pulse voltammetric determination of paracetamol using activated glassy carbon electrode. Am. J. Phys. Chem..

[32] Engin C., Yilmaz S., Saglikoglu G., Yagmur S., Sadikoglu M. (2015). Electroanalytical investigation of paracetamol on glassy carbon electrode by voltammetry. Int. J. Electrochem. Sci..

[33] Chitravathi S., Munichandraiah N. (2016). Voltammetric Determination of Paracetamol, Tramadol and caffeine using poly(nile blue) modified glassy carbon electrode. J. Electroanal. Chem..

[34] Delolo F.G., Rodrigues C., Silva M.M.d., Dinelli L.R., Delling F.N., Zukerman-Schpector J., Batista A.A. (2014). A new electrochemical sensor containing a film of chitosan-supported ruthenium: Detection and quantification of sildenafil citrate and acetaminophen. J. Braz. Chem. Soc..

[35] Grifasi N., Ziantoni B., Fino D., Piumetti M. (2024). Fundamental properties and sustainable applications of the natural zeolite clinoptilolite. Environ. Sci. Pollut. Res..

[36] Erdem E., Karapinar N., Donat R. (2004). The removal of heavy metal cations by natural zeolites. J. Colloid Interface Sci..

[37] Bahmanzadegan F., Ghaemi A. (2024). Modification and functionalization of zeolites to improve the efficiency of CO_2_ adsorption: A review. Case Stud. Chem. Environ. Eng..

[38] Akyalcin S., Akyalcin L., Ertugrul E. (2024). Modification of natural clinoptilolite zeolite to enhance its hydrogen adsorption capacity. Res. Chem. Intermed..

[39] Li Z., Bowman R.S. (1998). Sorption of perchloroethylene by surfactant-modified zeolite as controlled by surfactant loading. Environ. Sci. Technol..

[40] Dávila-Estrada M., Ramírez-García J.J., Solache-Ríos M.J., Gallegos-Pérez J.L. (2018). Kinetic and equilibrium sorption studies of ceftriaxone and paracetamol by surfactant-modified zeolite. Water Air Soil Pollut..

[41] Mozgawa W., Król M., Bajda T. (2011). IR spectra in the studies of anion sorption on natural sorbents. J. Mol. Struct..

[42] Verma Y., Sharma G., Kumar A., Wang T., Dhiman P., Tessema Mola G. (2025). Zeolites and their composites as novel remediation agent for antibiotics: A review. Environ. Eng. Res..

[43] Porada R., Wenninger N., Bernhart C., Fendrych K., Kochana J., Baś B., Kalcher K., Ortner A. (2023). Targeted modification of the carbon paste electrode by natural zeolite and graphene oxide for the enhanced analysis of paracetamol. Microchem. J..

[44] Smiljanić D., Daković A., Obradović M., Ožegović M., Izzo F., Germinario C., de Gennaro B. (2021). Application of surfactant modified natural zeolites for the removal of salicylic acid—A contaminant of emerging concern. Materials.

[45] Doczekalska B., Kuśmierek K., Świątkowski A. (2025). The adsorptive removal of paracetamol as a model pollutant from an aqueous environment using activated carbons made from selected nutshells as agricultural waste. Processes.

[46] Iancu V.-I., Chiriac L.-F., Paun I., Dinu C., Pirvu F., Cojocaru V., Tenea A.G., Cimpean I.A. (2025). Pharmaceutical contaminants occurrence and ecological risk assessment along the Romanian Black Sea coast. Toxics.

[47] Baerlocher C.h., Brouwer D., Marler B., McCusker L.B. Database of Zeolite Structures. https://www.iza-structure.org/databases/.

[48] Barczyk K., Mozgawa W., Król M. (2014). Studies of anions sorption on natural zeolites. Spectrochim. Acta A.

[49] Haro N.K., Dávila I.V.J., Nunes K.G.P., Espina de Franco M.A., Marcilio N.R., Féris L.A. (2021). Kinetic, equilibrium and thermodynamic studies of the adsorption of paracetamol in activated carbon in batch model and fixed-bed column. Appl. Water Sci..

[50] Aydin S., Celik Karakaya M., Karakaya N., Aydin M.E. (2023). Effective removal of selected pharmaceuticals from sewerage treatment plant effluent using natural clay (Na-montmorillonite). Appl. Water Sci..

